# Treating with Epidermal Growth Factor Receptor (EGFR) Tyrosine Kinase Inhibitors (TKIs) Accompanying Lower Incidence of Second Primary Cancers

**DOI:** 10.3390/jcm11175222

**Published:** 2022-09-04

**Authors:** Wen-Ru Chou, Ben-Chang Shia, Yen-Chun Huang, Chieh-Wen Ho, Mingchih Chen

**Affiliations:** 1 Department of Internal Medicine, Fu Jen Catholic University Hospital, Fu Jen Catholic University, New Taipei City 242062, Taiwan; 2 Graduate Institute of Business Administration, College of Management, Fu Jen Catholic University, No.510, Zhongzheng Rd., Xinzhuang Dist., New Taipei City 242062, Taiwan; 3 Artificial Intelligence Development Center, Fu Jen Catholic University, No.510, Zhongzheng Rd., Xinzhuang Dist., New Taipei City 242062, Taiwan; 4 Department of Life Science, National Taiwan University, Taipei 10617, Taiwan

**Keywords:** lung cancer, second primary cancers, risk factors, tyrosine kinase inhibitor

## Abstract

Lung cancer survivors are at risk of developing second primary cancers (SPCs). Although some risk factors for the development of SPCs have been addressed, their impacts have not been clarified. This study, based on Taiwan’s National Health Insurance Research Database (NHIRD), a nationwide database, was designed to investigate the risk factors for SPCs in patients with initial lung cancer and identify the impacts of epidermal growth factor receptor (EGFR) tyrosine kinase inhibitor (TKI) treatment on the development of SPCs. In this study, 37,954 individuals were included, of whom 2819 had SPCs. These patients were further divided into the second primary lung cancers (SPLC) and second primary extrapulmonary cancer (SPEC) groups. Among the patients with lung cancer without SPCs, those aged <65 years accounted for 53.15%. Patients aged ≥65 years accounted for 40.18% and 53.24% in the SPLC and SPEC groups, respectively. Females accounted for 50.3% of patients without SPC, 54% of the SPLC group, and 44.3% of the SPEC group. Univariate and multivariate Cox proportional hazard models showed increased hazard ratios for smoking, hypertension, and diabetes mellitus, and lower HRs for surgery, chemotherapy, radiotherapy, and TKIs. Patients undergoing surgery, chemotherapy, and radiotherapy were associated with a lower risk of SPCs. Treatment with EGFR TKIs was a significant and independent factor associated with lower incidence of SPCs. This study may encourage researchers to establish predictive models based on our results to assess the risk factors for SPCs, and therefore, early screening and intervention could be applied, and the SPCs-related mortality and relevant medical costs could be reduced.

## 1. Introduction

Recently, the development of chest computed tomography has improved the early diagnosis of lung cancer [1], and precision medicine with individualized and molecularly targeted therapy has improved the survival of patients with lung cancer [2]. Evidence shows that cancer survivors are at an increased risk of second primary cancers (SPCs) [3,4], and the incidence of SPCs has increased over time with a 10-year cumulative risk reaching as high as 13% for patients diagnosed with cancer at the age between 60 and 69 years [5]. A study used the Surveillance, Epidemiology, and End Results (SEER) 13 registry to examine the multiple primary standardized incidence ratio and risk between January 2004 and December 2010 for patients with stage Ia non-small cell lung cancer (NSCLC), and the results showed that 1431 (11.68%) of 12,246 patients had SPCs [6]. Another study demonstrated that the incidence of SPCs in patients diagnosed with NSCLC was 6.4%, while second primary lung cancers (SPLCs) were the leading type of SPC (45.1%) [7]. SPLC refers to new primary lung cancer that develops after an IPLC and second primary extrapulmonary cancer (SPEC) refers to a new extra-pulmonary primary cancer following an IPLC. The most frequent concomitant malignancies varied among different genders and countries. A previous study demonstrated that the most frequent concomitant malignancies in male patients involving lung cancer were gastric cancer, prostate cancer, and colon cancer, while in female patients, the most frequent concomitant malignancies were breast cancer, thyroid cancer, and colon cancer [8]. In the same database, our previous research revealed that the most common second primary malignancies following an IPLC were lung, colon, breast, and prostate cancers.

Some risk factors for SPCs have been addressed; however, some controversy remains. Several studies have demonstrated that men were more likely to have SPCs than women [9]. The age of initial primary cancer (IPC) diagnosis, sex, race, marital status, IPC tumor site, tumor size, TNM stage, extent of disease, and surgical history were the significant risk factors in predicting the risk of SPCs [10]. Numerous studies have emphasized that smoking was a significant risk factor for the development of SPCs [9,11,12,13,14]. The SEER data from 1992 to 2008 showed that 25,472 out of 1,450,837 survivors of non-pulmonary cancers had SPLCs at a mean follow-up period of 5.7 years and that 57% of the patients with SPLCs died of the disease. Increasing age and being divorced/widowed/separated were independent risk factors for the development of SPLC [15]. In addition to the baseline characteristics of IPC, treatments for IPC also affect the incidence of SPCs. A study demonstrated that patients who underwent radiotherapy and chemotherapy had more multiple primary cancers (MPCs) than those who never received these treatments [16]. In contrast, a study involving individuals with initial primary lung cancer (IPLC) who survived 2 years or more from the SEER-18 database in the 2004–2007 period revealed that IPLC survivors who received radiotherapy had a lower 5-year incidence of metachronous SPLCs than those who did not. A few studies have focused on the association of the risk of SPCs with other treatments for IPLCs, except for radiotherapy. A study implied that the use of tyrosine kinase inhibitors (TKIs) was associated with a significantly reduced risk of the development of SPCs [17]. Therefore, we extracted data from the National Health Insurance (NHI) Research Database (NHIRD) to specify several potential risk factors for SPCs and performed an inter-institution analysis. We separated some factors that may influence the incidence of SPCs. Furthermore, some risk factors such as smoking are risk factors for developing both SPLC and SPEC such as cancers of the mouth, pharynx, esophagus, larynx, and urinary tract [9]. The impacts of the risk factors for SPLC and SPEC need to be investigated.

## 2. Materials and Methods

### 2.1. Data Sources and Research Samples

The NHI was established in 1995 and covers approximately 90% of individuals in Taiwan who joined the program. The NHIRD provides plenty of medical information such as outpatient, emergency, and in-patient medical records. Aside from the medical records, the Taiwan Cancer Registry, one of the databases with the highest credibility, provides comprehensive data including the patients’ social behavior, baseline characteristics, cancer diagnoses, and treatments. This registry includes stages, tumor sizes, and histological types, and uses the third edition of the International Classification of Diseases for Oncology (ICD-O-3) to classify and stage cancers.

Since 2011, the Taiwan Cancer Registry (TCDB) has also provided more detailed information such as clinical data, lifestyle, height, and body weight. We enrolled the patient with lung cancer diagnosed from 2011 to 2016 and followed them until the end of the study, 31 December 2019, to investigate whether a second primary cancer occurred. Based on our previous study, the mean time of occurrence of a metachronous second primary cancer after an IPLC was 2.35 years. Therefore, we designed the study period from 2011 to 2016 because it might take time, with a mean time of 2.35 years, for an IPLC to develop a SPC. In this study, our main targets were anonymized in the NHIRD; thus, this study was fully adopted and reviewed by the Ethics Committee and Institutional Review Board of Fu Jen Catholic University (IRB number: C108121), and the requirement for informed consent was waived.

### 2.2. Study Population and Exclusion Criteria

**Figure 1:**
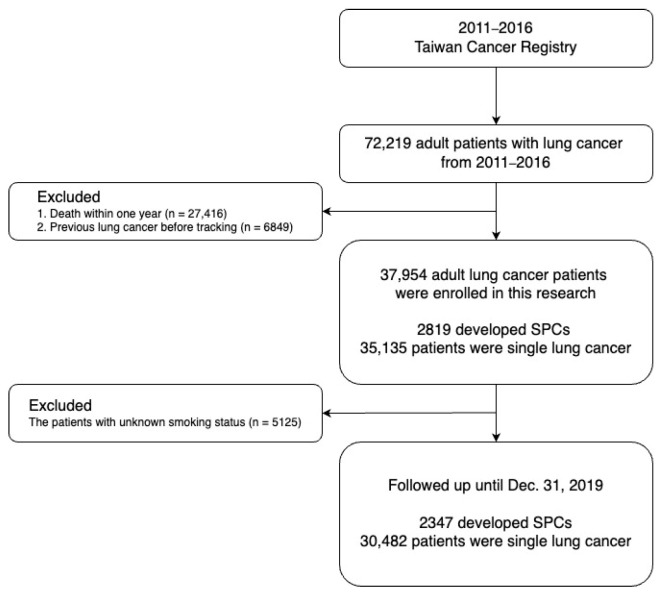
The study flowchart. SPCs—second primary cancers.

In this study, data on patients with lung cancer were extracted from the Taiwan Cancer Registry from 2011 to 2016 as the study subjects. This study was designed to determine the risk factors for developing SPCs and their association with the baseline characteristics or different treatments. The patients were divided into the following two groups: patients with and without SPCs, and those with SPCs were further divided into the SPLC and second primary extrapulmonary cancer (SPEC) groups. SPLC refers to a new primary lung cancer that develops after an IPLC, and SPEC refers to a new extra-pulmonary primary cancer following an IPLC. Patients aged less than 18 years and those with missing information were excluded from this study. The date of the diagnosis of lung cancer was the index date. The endpoint was the development of SPCs, and the end of follow-up was the date of death or the end of the follow-up period. Patients receiving surgical treatment, radiotherapy, epidermal growth factor receptor (EGFR) TKIs treatment, and chemotherapy during the follow-up period were included in this study (Figure 1). The first and second generation EGFR TKIs including gefitinib, erotinib, and afatinib were investigated in this study. In Taiwan, the National Health Insurance payment for the first EGFR TKI, gefitinib, was approved since 2004 and the third generation EGFR TKI, osimertinib, was approved since 2020.

### 2.3. Statistical Analysis

The chi-square test was used to examine the differences in the categorical variables, and the results are presented as numbers and percentages. The Student’s *t*-test was used to examine the continuous variables, and the results are presented as the means ± standard deviations. Furthermore, univariate and multivariate Cox proportional hazard (PH) models were used to adjust for confounding variables to determine the factors associated with the development of SPCs. The results of the correction are presented as adjusted hazard ratios (aHRs) and 95% confidence intervals (Cis). The Cox PH models were divided into two models. The first model used three variables (i.e., smoking status, EGFR mutation, and TKIs treatment). The second model applied all variables (i.e., baseline demographics and different treatments) to analyze the risk factors for the development of SPCs. All *p*-values were two-tailed, and *p*-values of less than 0.05 were used to denote statistical significance. All analyses in this study were performed using SAS software version 9.4 (SAS Institute Inc., Cary, NC, USA).

## 3. Results

Table 1 shows the baseline characteristics of the patients with lung cancer with and without SPC. Of the 37,954 individuals identified, 2819 had SPC, whereas 35,135 did not. Furthermore, those with SPCs were divided into the SPLC (*n* = 1339) and SPEC (*n* = 1480) groups. Among all of the individuals identified, most were aged <65 years, and of those without SPCs, 53.15% were aged <65 years. In the SPLC group, 40.18% of the patients were aged ≥65 years, while in the SPEC group, 53.24% of the patients were aged ≥65 years. The mean age of the patients without SPCs was 63.7 years. The mean age was 61.8 and 65.6 years in the SPLC and SPEC groups, respectively. Moreover, overall, females accounted for 50.3% of the patients without SPCs, whereas they accounted for 54% and 44.32% of the patients in the SPLC and SPEC groups, respectively.

Table 2 shows the comparisons of the age, sex, history of smoking, history of alcohol consumption, different cancer stages, EGFR mutation status, different histological types, body mass index, underlying diseases, and treatments for lung cancer in patients with and without SPCs. Of the patients without SPCs, 31.98% had a history of smoking, and 29.94% of patients with SPCs had a history of smoking (*p* < 0.001).

Moreover, of the patients without SPCs, 29.3% had hyperlipidemia, and 31.25% of patients without SPC had hyperlipidemia (*p* = 0.029). The proportion of patients with diabetes mellitus (DM) was significantly higher among patients with SPCs (21.92%) than among those without SPCs (20.38%) (*p* = 0.050); the proportion of patients with gout was significantly higher among patients with SPCs (10.71%) than among those without SPCs (9.56%) (*p* = 0.046). Significant differences in the treatments for lung cancer including surgery, chemotherapy, and radiotherapy were observed between the two groups. The proportion of patients receiving surgical treatments, radiotherapy, and chemotherapy was lower among patients with SPCs than among those without. The proportion of patients undergoing surgery was significantly higher among patients without SPCs (36.27%) than among those with SPCs (32.78%) (*p* < 0.001); that of patients receiving chemotherapy was significantly higher among patients without SPCs (17.75%) than among those with SPCs (2.66%) (*p* < 0.001); and that of patients receiving radiotherapy was significantly higher among patients without SPCs (26.15%) than among those with SPCs (4.04%) (*p* < 0.001).

As observed in Table 3, 54% of the patients in the SPLC group were female, and 55.68% of the patients in the SPEC group were male (*p* < 0.001). In the SPLC group, 59.82% of the patients were aged <65 years, whereas in the SPEC group, 53.24% of the patients were aged ≥65 years (*p* < 0.001). Significant differences in the history of smoking, history of alcohol consumption, EGFR mutation status, stages, histological type, body mass index, and underlying diseases including DM (SPLC: 18.75% vs. SPEC: 24.80%; *p* < 0.001), gout (SPLC: 9.19% vs. SPEC: 12.09%; *p* = 0.013), and COPD (SPLC: 28.45% vs. SPEC: 35.54%; *p* < 0.001) were observed between the SPLC and SPEC groups. Moreover, the proportion of patients who underwent surgery was significantly higher in the SPEC group (43.58%) than in the SPLC group (20.84%) (*p* < 0.001). Of the 1339 patients, four (0.3%) receiving chemotherapy and 20 (1.49%) receiving radiotherapy had SPLCs. All treatments showed significant differences (*p* < 0.001).

To identity the effects of smoking on the risk of developing SPCs, we excluded patients with unknown smoking history. The univariate and multivariate Cox PH models are shown in Table 4.

Table S1 shows the results of the multivariate analysis with the overall population. After excluding patients with unknown smoking status, the risk of developing SPCs during the follow-up period was evaluated using the univariate and multivariate Cox PH models. After adjusting for smoking history, EGFR mutation status, and TKIs treatment, Model 1 showed that smoking had a relatively high HR (aHR: 1.11; 95% confidence interval [CI]: 1.02–1.21; *p* = 0.015). EGFR mutation had an increased HR (aHR: 0.98; 95% CI: 0.85–1.12; *p* < 0.001), whereas TKIs had a significantly lower HR (aHR: 0.14; 95% CI: 0.10–0.19; *p* < 0.001). After adjusting for the overall variables, Model 2 showed that the early stages of lung cancer (stage 0, aHR: 1.69; stage 2, aHR: 1.24; *p* < 0.001), squamous cell type (aHR: 1.06; 95% CI: 0.92–1.24; *p* < 0.001), small cell type (aHR: 1.84; 95% CI: 1.50–2.25; *p* < 0.001), hypertension (aHR: 1.29; 95% CI: 1.12–1.49; *p* < 0.001), and DM (aHR: 1.13; 95% CI: 1.02–1.25; *p* = 0.022) were significantly associated with an increased risk of developing SPCs. Among the therapies, surgery (aHR: 0.34; 95% CI: 0.31–0.38; *p* < 0.001), chemotherapy (aHR: 0.23; 95% CI: 0.18–0.30; *p* < 0.001), radiotherapy (aHR: 0.24; 95% CI: 0.20–0.30; *p* < 0.001), and TKIs (aHR: 0.18; 95% CI: 0.13–0.24; *p* < 0.001) were significantly associated with a lower risk of developing SPCs. As observed in Table S2, male patients were at a higher risk of developing SPCs (aHR: 1.14; 95% CI: 1.04–1.25; *p* = 0.006).

## 4. Discussion

Cancer survival has significantly improved; however, patients with cancer with a longer lifespan are at a higher risk of developing SPCs. A study revealed that the risk of SPCs was higher in cancer survivors than in the general population, with a 3.8% higher probability of developing metachronous SPCs within a median follow-up period of 2.5 years, and that the incidence increased over time with a 10-year cumulative risk of SPCs reaching 13% for patients diagnosed with cancer between the ages of 60 and 69 years [5]. Therefore, lung cancer survivors have increased chances of developing SPCs. Some risk factors for the development of SPCs have been addressed; however, their impacts have not been clarified. Most studies that have explored the risk factors of SPCs focused on the basic characteristics of the patients and the IPC itself, and the influences of the underlying diseases and treatments were less addressed. Some risk factors associated with the baseline characteristics of the patients and treatments for IPCs remain controversial. In this study, we investigated the risk factors associated with the basic characteristics of patients including personal habits, underlying diseases, cell type, and stage of IPLCs. We further explored the risk factors of SPCs in subgroups with different treatments for lung cancer.

### 4.1. Risk Factors to the Baseline Characteristics

Some studies revealed that men are more likely to have SPCs than women. A recent study in Turkey reported that MPCs were detected more commonly in men than in women (*p* < 0.001) [16]. The same result was shown in a study in Taiwan [17]. In this study, similar to the results of previous studies, men were at a higher risk of developing SPCs with an HR of 1.14. Interestingly, in the subgroup analysis, a meta-analysis of 61 articles highlighted that night-shift work was associated with an increased risk of MPCs in women [18]. Another study revealed that smoking was a significant risk factor for the development of MPCs involving lung cancer [11]. A multi-institutional study in 1997 revealed that the risk of all SPCs (mostly NSCLC) among small cell lung cancer survivors was 3.5-fold compared with that in the general population. Smoking, radiotherapy, and chemotherapy also play a significant role in the development of SPCs [13]. The multivariate analysis in this study showed that smoking was associated with a higher risk of developing SPCS, with an HR of 1.113.

Another study examined the risk factors for SPLC with consistent results. Among the 7299 IPLC cases diagnosed from 1993 to 2017, 167 (2.3%) had SPLC, and smoking pack-years, smoking intensity, and individuals who met the screening criteria of the U.S. Preventative Services Task Force (i.e., aged 55–80 years, smoked ≥30 pack-years, and ≤15 years since smoking cessation) at the time of IPLC diagnosis were at a high risk of developing SPLC [14]. Moreover, smoking was emphasized as a risk factor for the development of cancers other than lung cancer. A large population-based cancer registry study in Germany confirmed that smoking increased the risk of tobacco-related subsequent primary cancer including cancers of the mouth/pharynx, esophagus, larynx, lung/bronchus, kidney, urinary bladder, and urinary tract. Between 2002 and 2008, 121,631 cases of tobacco-related first primary cancer in men and 75,886 in women were registered, and 2.5% male and 1.2% female patients with cancer had at least one tobacco-related subsequent primary cancer [9]. A retrospective study analyzed patients with NSCLC diagnosed between 2004 and 2010 in the SEER database and demonstrated that the crude and 10-year cumulative incidence rates of SPCs were 4.04% and 5.05%, respectively. The staff developed the first competing risk nomogram to predict the risks of SPMs. The age at IPC diagnosis, sex, race, marital status, IPC tumor site, tumor size, TNM stage, extent of disease, and surgical history were significant risk factors for SPCs. Tumor TNM stage, extent of disease, and surgical history were the most crucial factors for predicting SPC risk. Patients with advanced stages were less likely to develop SPCs, and conversely, patients with smaller tumor sizes and those whose tumors were located in the upper lung were more likely to have SPCs [10]. Hypertension (aHR: 1.29; 95% CI: 1.12–1.49; *p* < 0.001) and DM (aHR: 1.13; 95% CI: 1.02–1.25; *p* = 0.022) were significantly associated with an increased risk of developing SPCs. A study used the SEER-13 registry to examine the multiple primary standardized incidence ratio and absolute excess risk between January 2004 and December 2010 for patients with stage 1a NSCLC and showed that 1431 (11.68%) out of 12,246 patients had SPMs, with an observed to expected ratio of 2.07 (95% CI: 1.92–2.23; *p* < 0.001) in patients with adenocarcinoma and 2.05 (95% CI: 1.92–2.19; *p* < 0.001) in patients with squamous cell carcinoma [6]. This study demonstrated similar results, showing that the early stages of lung cancer (stages 0, 1, and 2) and squamous and small cell cancer types were associated with a higher risk of developing SPCS. Many studies have attempted to establish predictive models to assess the risk factors for SPLC, the goal being early screening and intervention, which could reduce the SPLC-related mortality. Another study estimated the 10-year risk of developing SPLCs among IPLC survivors using SEER data, examined the risk-stratification ability of the prediction model, and performed decision curve analysis. The median 10-year risk of SPLC among IPLC survivors was 8.36%, and the estimated risk varied substantially (range, 0.56–14.3%) when stratified according to age, histology, and the extent of IPLC in the final prediction model [19]. More patients with SPCs had liver cirrhosis and underwent surgery for lung cancer, whereas fewer patients with SPCs received chemotherapy, radiotherapy, and TKIs. Age ≥ 50 years, being male, and having liver cirrhosis were significantly associated with a higher risk of SPC [17]. In this study, by connecting to the NHIRD, we presented different findings; lung cancer patients with a history of hypertension and DM were significantly associated with an increased risk of developing SPCs according to the multivariate analysis performed in this study.

### 4.2. Treatment-Related Risk Factors

Regarding the treatment-related risk factors for SPCs, radiotherapy and chemotherapy for IPCs were addressed; however, the results were inconsistent. There is some debate about whether radiotherapy is associated with a higher risk of developing SPCs. Patients who received radiotherapy and chemotherapy had a higher risk of developing SPCs than those who never received these treatments [16]. Patients receiving radiotherapy (*p* < 0.001) and chemotherapy (*p* < 0.001) had more SPCs than those who never received these treatments. A group of scientists discovered that the incidence of SPCs was increased in patients with prostate cancer treated with radiotherapy compared to that in the general population [20]. In contrast, another group of scholars suggested that applying radiotherapy is a negative independent risk factor for developing metachronous SPLC [21]. The former study evaluated the characteristics of multiple malignancies and analyzed the risk of SPCs after radiotherapy for prostate cancer. The results showed that patients who received radiotherapy for prostate cancer were at a high risk of developing ureter cancers and malignant lymphoma [20]. The latter study estimated the incidence of metachronous SPLC in IPLC survivors and determined the radiotherapy-related risks; however, it presented different results. The study enrolled IPLC individuals who survived 2 years or more from the SEER-18 database in 2004–2007 and revealed that IPLC survivors treated with radiotherapy had a lower 5-year incidence of metachronous SPLC, particularly those with NSCLC, than those not treated with radiotherapy [21]. Some studies have analyzed the cumulative risk of multiple risk factors for SPCs. Patients who received chest irradiation had a 13-fold increased risk, and the relative risk was 21% in patients who had received chest irradiation and continued smoking. Furthermore, there was a 19-fold risk increase among current smokers treated with alkylating agents [13].

Among the patients with NSCLC, particularly those of Asian origin, a large proportion had EGFR mutations. In mainland China, the overall frequency of the EGFR mutations was 50.2% and 35.3% among the regular smokers, respectively [22]. The treatment for patients with advanced NSCLC with EGFR mutations is EGFR TKI therapy [22]. With the development of genetic testing, recent studies have begun to focus on genetic factors related to the development of SPCs. EGFR, KRAS, TP53, and PARP1 mutations were concomitantly detected in some individuals with multiple primary lung cancers. Unsurprisingly, EGFR mutations were significantly more frequent in women and in never or light smokers. Concomitant EGFR or KRAS mutations were particularly prominent in men and in never or light smokers [23]. In this study, we only investigated the expression of EGFR; no other mutation data such as for KRAS, TP53, or PARP1 mutations were examined. Except for radiotherapy and chemotherapy, the associated risk of TKI is an important issue in IPLC survivors. A population-based cohort study using the Registry of Catastrophic Illness enrolled patients with lung cancer diagnosed between 1997 and 2005 and followed them up until 31 December 2011, and the results showed that the use of TKIs was associated with a statistically significantly reduced incidence of SPCs (HR, 0.41; 95% CI, 0.21–0.79; *p* = 0.008). In contrast, surgery, chemotherapy, and radiotherapy showed no correlation [17]. Because the smoking status, pathological findings, and stages of lung cancer were unavailable in the study, the author emphasized that the results should be interpreted cautiously because TKIs users usually have adenocarcinoma, which is less associated with smoking, and therefore, these patients might have fewer smoking-related SPCs. The author also explained that the lower risk of SPMs among patients with lung cancer treated with TKIs may be related to the downregulation of EGFR activity.

In this study, we recorded the smoking status and developed univariate and multivariate Cox PH models by adjusting for smoking habit and EGFR status, and the results showed that the use of TKIs was an independent risk factor associated with a lower incidence of SPCs. Surgical treatment, chemotherapy, and radiotherapy were also associated with a lower risk of SPCs. To identify whether smoking or TKIs usage is an independent risk factor, we adjusted for smoking and EGFR. We excluded patients with unknown smoking status to understand the risk of SPC in smokers and non-smokers. Conversely, we did not exclude patients with unknown EGFR status for the following considerations. In this study, the patient with unknown EGFR status accounted for more than half of all patients. EGFR testing is now becoming more and more available and affordable, but not in 2016 or earlier. With the understanding of biomarkers for lung cancer, biomarker studies including EGFR expression are strongly recommended in advanced NSCLC, but not in all stages. In this study, 60% of patients diagnosed with stages I to III and these patients did not routinely receive EGFR testing. Our data reflected the real world practice.

In our study, the HRs of EGFR-TKIs, surgery, radiotherapy, and chemotherapy differed significantly in the incidence of developing SPC. While surgery, radiotherapy, and chemotherapy are performed alone or in combination at various stages for curative treatment, local control, adjuvant therapy, or palliative care, the use of EGFR-TKIs is specifically indicated and approved by Taiwan health insurance payments for EGFR-mutant advanced NSCLC. In fact, we performed subgroup analyses for each stage and the results were promising. However, some subgroups had small numbers of cases and need cumulating to be statistically analyzed. Further studies with a larger sample size, different stages, and treatment options could clarify the impact of each risk factor on the SPCs.

## 5. Conclusions

Male patients and those with early stages of lung cancer, squamous and small cell types, and a history of smoking are at a higher risk of developing SPCs. Surgical treatment, chemotherapy, and radiotherapy are associated with a lower risk of SPCs. Treatment with EGFR TKIs was a significant and independent factor associated with a lower incidence of SPCs. The limitation of this study was the lack of complete personal histories and biobank in this study; a small proportion of unknown personal habits may affect the case number and results. Another limitation was that the environmental factor was not included in the NHIRD. This study may encourage researchers to establish predictive models based on our results to assess the risk factors for SPCs, and therefore, early screening and intervention could be applied, and the SPC-related mortality and relevant medical costs could be reduced.

## Figures and Tables

**Table 1:** The baseline characteristics of patients with lung cancer with and without SPCs (SPLC and SPEC groups, respectively).

Characteristics		Total Lung Cancer (*n* = 37,954)	Lung Cancer without SPC (*n* = 35,135)	Lung Cancer with SPC (*n* = 2819)	
				SPLC (*n* = 1339)	SPEC (*n* = 1480)
		*n* (%)	*n* (%)	*n* (%)	*n* (%)
Sex	Female	19,039 (50.16)	17,660 (50.26)	723 (54.00)	656 (44.32)
	Male	18,915 (49.84)	17,475 (49.74)	616 (46.00)	824 (55.68)
Age	<65	20,166 (53.13)	18,673 (53.15)	801 (59.82)	692 (46.76)
	≥65	17,788 (46.87)	16,462 (46.85)	538 (40.18)	788 (53.24)
Age, mean (SD)		63.75 (12.2)	63.74 (12.2)	61.81 (11.7)	65.6 (11.3)
History of smoking	Yes	12,079 (31.83)	11,235 (31.98)	331 (24.72)	513 (34.66)
	No	20,750 (54.67)	19,247 (54.78)	725 (54.14)	778 (52.57)
	Unknown	5125 (13.50)	4653 (13.24)	283 (21.14)	189 (12.77)
History of alcohol consumption	Yes	6030 (15.89)	5589 (15.91)	154 (11.50)	287 (19.39)
	No	25,857 (68.13)	24,028 (68.39)	872 (65.12)	957 (64.66)
	Unknown	6067 (15.99)	5518 (15.71)	313 (23.38)	236 (15.95)
EGFR	Yes	11,953 (31.49)	11,385 (32.40)	294 (21.96)	274 (18.51)
	No	5757 (15.17)	5424 (15.44)	206 (15.38)	127 (8.58)
	Unknown	20,244 (53.34)	18,326 (52.16)	839 (62.66)	1079 (72.91)
Stage of lung cancer	0	680 (1.79)	566 (1.61)	42 (3.14)	72 (4.86)
	1	10,623 (27.99)	9515 (27.08)	563 (42.05)	545 (36.82)
	2	2294 (6.04)	2057 (5.85)	69 (5.15)	168 (11.35)
	3	5674 (14.95)	5301 (15.09)	156 (11.65)	217 (14.66)
	4	15,315 (40.35)	14,711 (41.87)	302 (22.55)	302 (20.41)
	Unknown	3368 (8.87)	2985 (8.50)	207 (15.46)	176 (11.89)
Histological type of lung cancer	AC	29,842 (78.63)	27,758 (79)	1020 (76.18)	1064 (71.89)
	SCC	4228 (11.14)	3907 (11.12)	94 (7.02)	227 (15.34)
	Small cell	1916 (5.05)	1737 (4.94)	125 (9.34)	54 (3.65)
	Others	1968 (5.19)	1733 (4.93)	100 (7.47)	135 (9.12)
BMI	Under weight	4697 (12.38)	4368 (12.43)	160 (11.95)	169 (11.42)
	Normal	13,849 (36.49)	12,871 (36.63)	432 (32.26)	546 (36.89)
	Oberg weight	12,943 (34.10)	12,003 (34.16)	412 (30.77)	528 (35.68)
	Unknown	6465 (17.03)	5893 (16.77)	335 (25.02)	237 (16.01)
Underlying disease	Hypertension	3807 (10.03)	3535 (10.06)	131 (9.78)	141 (9.53)
	Hyperlipidemia	11,176 (29.45)	10,295 (29.3)	410 (30.62)	471 (31.82)
	DM	7777 (20.49)	7159 (20.38)	251 (18.75)	367 (24.8)
	CHF	2629 (6.93)	2447 (6.96)	77 (5.75)	105 (7.09)
	Stroke	3572 (9.41)	3325 (9.46)	105 (7.84)	142 (9.59)
	Gout	3660 (9.64)	3358 (9.56)	123 (9.19)	179 (12.09)
	COPD	12,077 (31.82)	11,170 (31.79)	381 (28.45)	526 (35.54)
Treatment	Operation	13,669 (36.01)	6235 (17.75)	279 (20.84)	645 (43.58)
	Chemotherapy	6310 (16.63)	12,745 (36.27)	4 (0.30)	71 (4.80)
	Radiotherapy	9301 (24.51)	9187 (26.15)	20 (1.49)	94 (6.35)

SPCs—second primary cancers; SPLC—second primary lung cancer; SPEC—second primary extrapulmonary cancer; AC—adenocarcinoma; SCC—squamous cell carcinoma; SD—standard deviation; BMI—body mass index; EGFR—epidermal growth factor receptor; DM—diabetes mellitus; CHF—congestive heart failure; COPD—chronic obstructive pulmonary disease.

**Table 2:** A comparison of the characteristics between patients with lung cancer with and without SPCs.

Characteristics		Lung Cancer without SPC (*n* = 35,135)	Lung Cancer with SPC (*n* = 2819)	*p*
		*n* (%)	*n* (%)	
Sex	Female	17,660 (50.26)	1379 (48.92)	0.169
	Male	17,475 (49.74)	1440 (51.08)	
Age	<65	18,673 (53.15)	1493 (52.96)	0.850
	≥65	16,462 (46.85)	1326 (47.04)	
Age, mean (SD)		63.7 (12.2)	63.8 (11.6)	0.770
History of smoking	Yes	11,235 (31.98)	844 (29.94)	<0.001
	No	19,247 (54.78)	1503 (53.32)	
	Unknown	4653 (13.24)	472 (16.74)	
History of alcohol consumption	Yes	5589 (15.91)	441 (15.64)	<0.001
	No	24,028 (68.39)	1829 (64.88)	
	Unknown	5518 (15.71)	549 (19.47)	
EGFR	Yes	11,385 (32.40)	294 (21.96)	<0.001
	No	5424 (15.44)	206 (15.38)	
	Unknown	18,326 (52.16)	839 (62.66)	
Stage of lung cancer	0	566 (1.61)	114 (4.04)	<0.001
	1	9515 (27.08)	1108 (39.30)	
	2	2057 (5.85)	237 (8.41)	
	3	5301 (15.09)	373 (13.23)	
	4	14,711 (41.87)	604 (21.43)	
	Unknown	2985 (8.50)	383 (13.59)	
Histological type of lung cancer	AC	27,758 (79.00)	2084 (73.93)	<0.001
	SCC	3907 (11.12)	321 (11.39)	
	Small cell	1737 (4.94)	179 (6.35)	
	Others	1733 (4.93)	235 (8.34)	
BMI	Under weight	4368 (12.43)	329 (11.67)	<0.001
	Normal	12,871 (36.63)	978 (34.69)	
	Oberg weight	12,003 (34.16)	940 (33.35)	
	Unknown	5893 (16.77)	572 (20.29)	
Underlying disease	Hypertension	3535 (10.06)	272 (9.65)	0.483
	Hyperlipidemia	10,295 (29.3)	881 (31.25)	0.029
	DM	7159 (20.38)	618 (21.92)	0.050
	CHF	2447 (6.96)	182 (6.46)	0.306
	Stroke	3325 (9.46)	247 (8.76)	0.220
	Gout	3358 (9.56)	302 (10.71)	0.046
Treatment	Operation	12,745 (36.27)	924 (32.78)	<0.001
	Chemotherapy	6235 (17.75)	75 (2.66)	<0.001
	Radiotherapy	9187 (26.15)	114 (4.04)	<0.001

**Table 3:** A comparison of the characteristics between the SPLC and SPEC groups.

Characteristics		SPLC (*n* = 1339)	SPEC (*n* = 1480)	*p*
		*n* (%)	*n* (%)	
Sex	Female	723 (54.00)	656 (44.32)	<0.001
	Male	616 (46.00)	824 (55.68)	
Age	<65	801 (59.82)	692 (46.76)	<0.001
	≥65	538 (40.18)	788 (53.24)	
Age, mean (SD)		61.8 (11.7)	65.6 (11.3)	<0.001
History of smoking	Yes	331 (24.72)	513 (34.66)	<0.001
	No	725 (54.14)	778 (52.57)	
	Unknown	283 (21.14)	189 (12.77)	
History of alcohol consumption	Yes	154 (11.50)	287 (19.39)	<0.001
	No	872 (65.12)	957 (64.66)	
	Unknown	313 (23.38)	236 (15.95)	
EGFR	Yes	294 (21.96)	274 (18.51)	<0.001
	No	206 (15.38)	127 (8.58)	
	Unknown	839 (62.66)	1079 (72.91)	
Stage of lung cancer	0	42 (3.14)	72 (4.86)	<0.001
	1	563 (42.05)	545 (36.82)	
	2	69 (5.15)	168 (11.35)	
	3	156 (11.65)	217 (14.66)	
	4	302 (22.55)	302 (20.41)	
	Unknown	207 (15.46)	176 (11.89)	
Histological type of lung cancer	AC	1020 (76.18)	1064 (71.89)	<0.001
	SCC	94 (7.02)	227 (15.34)	
	Small cell	125 (9.34)	54 (3.65)	
	Others	100 (7.47)	135 (9.12)	
BMI	Under weight	160 (11.95)	169 (11.42)	<0.001
	Normal	432 (32.26)	546 (36.89)	
	Oberg weight	412 (30.77)	528 (35.68)	
	Unknown	335 (25.02)	237 (16.01)	
Underlying disease	Hypertension	131 (9.78)	141 (9.53)	0.818
	Hyperlipidemia	410 (30.62)	471 (31.82)	0.491
	DM	251 (18.75)	367 (24.8)	<0.001
	CHF	77 (5.75)	105 (7.09)	0.147
	Stroke	105 (7.84)	142 (9.59)	0.100
	Gout	123 (9.19)	179 (12.09)	0.013
	COPD	381 (28.45)	526 (35.54)	<0.001
Treatment	Operation	279 (20.84)	645 (43.58)	<0.001
	Chemotherapy	4 (0.30)	71 (4.80)	<0.001
	Radiotherapy	20 (1.49)	94 (6.35)	<0.001

**Table 4:** The univariate and multivariate Cox proportional hazard models after excluding patients with unknown smoking status.

**Model 1**					
**Characteristics**		**Univariate Analysis**	***p***	**Multivariate Analysis**	***p***
History of smoking	No (ref)	1	<0.001	1	0.015
	Yes	1.16 (1.07–1.26)		1.11 (1.02–1.21)	
EGFR	No (ref)	1	<0.001	1	<0.001
	Yes	0.81 (0.71–0.93)		0.98 (0.85–1.12)	
	Unknown	1.24 (1.10–1.39)		1.20 (1.07–1.36)	
TKIs		0.13 (0.09–0.18)	<0.001	0.14 (0.10–0.19)	<0.001
**Model 2**					
**Characteristics**		**Univariate Analysis**	***p***	**Multivariate Analysis**	***p***
Age	<65 (ref)	1	<0.001	1	0.215
	≥65	1.20 (1.10–1.30)		1.06 (0.97–1.15)	
Sex	Female (ref)	1	<0.001	1	0.112
	Male	1.23 (1.14–1.34)		1.09 (0.98–1.22)	
History of smoking	No (ref)	1	<0.001	1	0.700
	Yes	1.16 (1.07–1.26)		1.02 (0.91–1.16)	
History of alcohol consumption	No (ref)	1	0.007	1	0.250
	Yes	1.13 (1.02–1.26)		1.10 (0.97–1.23)	
	Unknown	1.31 (1.05–1.64)		1.11 (0.88–1.40)	
EGFR	No (ref)	1	<0.001	1	0.424
	Yes	0.81 (0.71–0.93)		0.97 (0.85–1.12)	
	Unknown	1.24 (1.10–1.39)		1.05 (0.92–1.19)	
Stage of lung cancer	0	1.99 (1.64–2.43)	<0.001	1.69 (1.38–2.07)	<0.001
	1	1		1	
	2	1.18 (1.01–1.36)		1.24 (1.07–1.45)	
	3	0.97 (0.86–1.09)		0.97 (0.85–1.11)	
	4	0.76 (0.68–0.84)		0.75 (0.66–0.85)	
	Unknown	2.74 (2.20–3.40)		2.09 (1.66–2.63)	
Histological type of lung cancer	AC (ref)	1	<0.001	1	<0.001
	SCC	1.31 (1.15–1.49)		1.06 (0.92–1.24)	
	Small cell	1.79 (1.49–2.15)		1.84 (1.50–2.25)	
	Others	1.67 (1.43–1.96)		1.16 (0.98–1.37)	
BMI	Under weight	1.00 (0.88–1.13)	0.157	0.97 (0.86–1.1)	0.589
	Normal	1		1	
	Oberg weight	0.95 (0.87–1.04)		0.94 (0.86–1.03)	
	Unknown	1.19 (0.98–1.44)		0.92 (0.75–1.10)	
Hypertension		1.34 (1.17–1.54)	<0.001	1.29 (1.12–1.49)	<0.001
DM		1.21 (1.10–1.33)	<0.001	1.13 (1.02–1.25)	0.022
Hyperlipidemia		1.07 (0.98–1.16)	0.159	1.01 (0.92–1.11)	0.785
CHF		1.10 (0.94–1.30)	0.240	0.89 (0.74–1.05)	0.172
Stroke		1.09 (0.94–1.25)	0.262	0.96 (0.83–1.11)	0.601
Gout		1.23 (1.08–1.40)	0.002	1.10 (0.96–1.26)	0.167
COPD		1.05 (0.96–1.14)	0.308	0.96 (0.88–1.05)	0.358
Operation		0.48 (0.44–0.52)	<0.001	0.34 (0.31–0.38)	<0.001
Chemotherapy		0.18 (0.14–0.23)	<0.001	0.23 (0.18–0.30)	<0.001
Radiotherapy		0.21 (0.18–0.26)	<0.001	0.24 (0.20–0.30)	<0.001
TKIs		0.13 (0.09–0.18)	<0.001	0.18 (0.13–0.24)	<0.001

TKIs—tyrosine kinase inhibitors.

## Data Availability

Not applicable.

## Supplementary Materials

The following supporting information can be downloaded at: https://www.mdpi.com/article/10.3390/jcm11175222/s1, Table S1: Univariate and multivariate Cox proportional hazard models in the overall study population; Table S2: Univariate and multivariate Cox proportional hazard models in the overall study population after adjusting for all variables.
