# Supplementary material for: Treating with Epidermal Growth Factor Receptor (EGFR) Tyrosine Kinase Inhibitors (TKIs) Accompanying Lower Incidence of Second Primary Cancers
Source: J Clin Med. 2022 Sep 4;11(17):5222. doi: 10.3390/jcm11175222 (PMC9457496; doi:10.3390/jcm11175222)
Supplement: Supplementary file 1 [file jcm-11-05222-s001.zip › jcm-1864053-supplementary.pdf]

Table S1. Univariate and multivariate Cox proportional hazard models in the overall study population.

Characteristics		Overall			
		**Univariate analysis**	**p**	**Multivariate analysis**	**p**
History of smoking	No (ref)	1		1	
	Yes	1.16 (1.07–1.27)	<0.001	1.11 (1.02–1.21)	<0.001
	Unknown	1.59 (1.43–1.76)		1.50 (1.35–1.67)	
EGFR	No (ref)	1		1	
	Yes	0.81 (0.71–0.92)	<0.001	0.98 (0.85–1.12)	<0.001
	Unknown	1.32 (1.17–1.48)		1.21 (1.08–1.36)	
TKI		0.11 (0.08–0.15)	<0.001	0.12 (0.09–0.16)	<0.001
EGFR, epidermal growth factor recepto					

Table S2. Univariate and multivariate Cox proportional hazard models in the overall study population after adjusting for all variables.

Characteristics		Overall			
		**Univariate analysis**	**p**	**Multivariate analysis**	**p**
Age	<65 (ref)	1	<0.001	1	0.812
	≥65	1.21 (1.12–1.30)		1.01 (0.93–1.09)	
Sex	Female (ref)	1	<0.001	1	0.006
	Male	1.22 (1.14–1.32)		1.139 (1.04–1.25)	
History of smoking	No (ref)	1		1	
	Yes	1.16 (1.07–1.27)	<0.001	0.99 (0.88–1.11)	0.357
	Unknown	1.59 (1.43–1.76)		0.82 (0.62–1.08)	
History of alcohol consumption	No (ref)	1		1	
	Yes	1.13 (1.02–1.26)	<0.001	1.09 (0.972–1.23)	0.222
	Unknown	1.53 (1.39–1.68)		1.14 (0.91–1.421)	
EGFR	No (ref)	1		1	
	Yes	0.81 (0.71–0.92)	<0.001	0.97 (0.85–1.12)	0.192
	Unknown	1.32 (1.17–1.48)		1.07 (0.95–1.22)	
Stage of lung cancer	0	2.04 (1.68–2.48)		1.72 (1.41–2.09)	
	1	1		1	
	2	1.21 (1.05–1.39)	<0.001	1.27 (1.10–1.47)	<0.001
	3	0.98 (0.87–1.10)		0.98 (0.86–1.11)	
	4	0.79 (0.71–0.87)		0.80 (0.71–0.91)	
	Unknown	1.74 (1.55–1.96)		1.55 (1.31–1.82)	
Histological type of lung cancer	AC (ref)	1		1	
	SCC	1.32 (1.17–1.49)	<0.001	1.06 (0.93–1.22)	<0.001
	Small cell	2.16 (1.85–2.52)		2.14 (1.81–2.53)	

	Others	1.62 (1.41–1.85)		1.03 (0.90–1.19)	
BMI	Under weight	1.00 (0.88–1.13)	<0.001	0.98 (0.86–1.11)	0.584
	Normal	1		1	
	Oberg weight	0.95 (0.87–1.04)		0.94 (0.86–1.03)	
	Unknown	1.45 (1.32–1.61)		1.02 (0.85–1.23)	
Hypertension		1.34 (1.18–1.52)	<0.001	1.31 (1.15–1.50)	<0.001
Diabetes mellitus		1.18 (1.08–1.29)	<0.001	1.12 (1.02–1.23)	0.024
Hyperlipidemia		1.06 (0.98–1.15)	0.145	1.03 (0.95–1.12)	0.471
CHF		1.07 (0.92–1.24)	0.385	0.85 (0.72–1.00)	0.045
Stroke		1.10 (0.96–1.25)	0.157	0.97 (0.85–1.11)	0.641
Gout		1.21 (1.08–1.37)	0.002	1.09 (0.96–1.23)	0.168
COPD		1.07 (0.99–1.16)	0.078	1.00 (0.92–1.08)	0.946
Operation		0.47 (0.43–0.51)	<0.001	0.35 (0.32–0.39)	<0.001
Chemotherapy		0.18 (0.14–0.22)	<0.001	0.23 (0.18–0.29)	<0.001
Radiotherapy		0.19 (0.16–0.23)	<0.001	0.22 (0.18–0.26)	<0.001
TKI		0.11 (0.08–0.15)	<0.001	0.15 (0.11–0.21)	<0.001

AC, adenocarcinoma; SCC, squamous cell carcinoma; SD, standard deviation; BMI, body mass index; EGFR, epidermal growth factor receptor; DM, diabetes mellitus; CHF, congestive heart failure; COPD, chronic obstructive pulmonary disease.
